# *In vitro* Intestinal Mucosal Epithelial Responses to Wild-Type *Salmonella* Typhi and Attenuated Typhoid Vaccines

**DOI:** 10.3389/fimmu.2013.00017

**Published:** 2013-02-12

**Authors:** Maria Fiorentino, Karen M. Lammers, Myron M. Levine, Marcelo B. Sztein, Alessio Fasano

**Affiliations:** ^1^Department of Pediatrics, Mucosal Biology Research Center, University of Maryland School of MedicineBaltimore, MD, USA; ^2^Department of Pediatrics, Center for Vaccine Development, University of Maryland School of MedicineBaltimore, MD, USA

**Keywords:** intestinal mucosal barrier, epithelial permeability, mucosal immunity, *Salmonella* Typhi, typhoid vaccines, cytokines

## Abstract

Typhoid fever, caused by *S*. Typhi, is responsible for approximately 200,000 deaths per year worldwide. Little information is available regarding epithelium-bacterial interactions in *S*. Typhi infection. We have evaluated *in vitro* the effects of wild-type *S*. Typhi, the licensed Ty21a typhoid vaccine and the leading strains CVD 908-*htrA* and CVD 909 vaccine candidates on intestinal barrier function and immune response. Caco2 monolayers infected with wild-type *S*. Typhi exhibited alterations in the organization of tight junctions, increased paracellular permeability, and a rapid decrease in Trans-Epithelial Electrical Resistance as early as 4 h post-exposure. *S*. Typhi triggered the secretion of interleukin (IL)-8 and IL-6. Caco2 cells infected with the attenuated strains exhibited a milder pro-inflammatory response with minimal disruption of the barrier integrity. We conclude that wild-type *S*. Typhi causes marked transient alterations of the intestinal mucosa that are more pronounced than those observed with Ty21a or new generation attenuated typhoid vaccine candidates.

## Introduction

*Salmonella* spp. are highly invasive pathogens. *Salmonella enterica* serovar Typhi (*S*. Typhi) and *S*. Paratyphi A, B, and C are the causing agents of enteric fevers. Typhoid fever is an acute, life-threatening febrile illness caused by *S*. Typhi which results in ∼200,000 deaths worldwide each year, largely in developing nations (Crump et al., 2004). Typhoid fever also occurs among persons living in industrialized countries, many belonging to known risk groups, such as travelers to endemic regions, including military personnel. Humans are the only reservoir for *S*. Typhi and infection occurs through ingestion of contaminated food or water. Following ingestion, the bacteria spread from the intestine via the blood where they multiply to the intestinal lymph nodes, liver, and spleen. Typhoid fever is characterized by fever and abdominal symptoms. In 5–10% of infected people, neuropsychiatric manifestations occur. Complications such as gastrointestinal bleeding, intestinal perforation, and typhoid encephalopathy occur in 10–15% of patients (Fraser et al., 2007).

The first significant cellular contact enteric pathogens have with the host, occurs at the level of the intestinal epithelium. Invasive bacteria have evolved an array of mechanisms to breach the integrity of the intestinal epithelial barrier, either by targeting tight junction proteins directly or by altering transduction signals regulating their assembly (Fasano et al., 1995; Aktories, 1997; Wu et al., 1998; Pothoulakis, 2000; Simonovic et al., 2000). In most cases, however it is unclear if these cellular events represent a direct effect of bacterial mediators or compensatory responses of the host epithelial cells.

Due to the high burden of typhoid fever and increasing antibiotic resistance, vaccine development remains a high priority. Neither of the two vaccines currently available for the prevention of typhoid fever, including the orally administered Ty21a, is completely effective, with protection rates ranging between 60 and 80% (Levine et al., 1999, 2007; Guzman et al., 2006). Attenuated, oral typhoid vaccine candidate strains harboring *aro* mutations have been evaluated in volunteers (Hone et al., 1992) and some of them, such as CVD 908-*htrA* and its derivative CVD 909, have been shown to be well tolerated and highly immunogenic in clinical trials (Tacket et al., 2000a,b, 2004; Salerno-Goncalves et al., 2003, 2004; Sztein, 2007; Wahid et al., 2007, 2008, 2011).

Most of the studies aimed to evaluate the effect of *Salmonella* spp. on epithelial cells have been focused on *S*. Typhimurium or other non-typhoid spp. and demonstrated the ability of *Salmonella* to disrupt barrier integrity by modulating epithelial permeability, as indicated by a reduction in Trans-Epithelial Electric Resistance (TEER) and alteration of tight junction expression and/or distribution (Finlay et al., 1988; Finlay and Falkow, 1990; Jones et al., 1994; Clark et al., 1998; Jepson et al., 2000; Bertelsen et al., 2004; Otte and Podolsky, 2004; Kohler et al., 2007). Very limited information is available regarding *S*. Typhi’s ability to disrupt the epithelial barrier and no studies so far have described the interaction of *aro* mutants with human enterocytes. To our knowledge, only one study demonstrated that *S*. Typhi is able to disrupt the epithelial barrier *in vitro* by decreasing TEER in Hep-2 and Caco2 cell monolayers (Solano et al., 2001). Despite the wealth of available information on the pathogenesis of *Salmonella* spp., a systematic study of the effects of *S*. Typhi or attenuated *S*. Typhi vaccines on the integrity and function of human epithelial cells has never been performed. To this end, we have exploited a human cellular intestinal model system to evaluate the epithelial events occurring at the host-bacterial interface following infection with wild-type *S*. Typhi, the licensed attenuated oral Ty21a typhoid vaccine and the leading strains CVD 908-*htrA* and CVD 909 typhoid vaccine candidates.

## Materials and Methods

### Cell culture

Human Caco2 intestinal epithelial cells [HTB-37, American Type Culture Collection (ATCC), Rockville, MD, USA] were grown in Dulbecco’s Modified Eagle Medium (DMEM) supplemented with fetal bovine serum, glutamine, and antibiotics. Caco2 cells are polarized epithelial cells that form apical junctional complexes, resulting in high electrical resistance, useful for studying effects of bacteria on permeability. Monolayers (passages 22–30) were grown on 1.12 cm^2^ permeable polyester filters with 0.4 μm pore size (Corning, Lowell, MA, USA) and utilized after 14–21 days until having reached a confluent, polarized, and differentiated state. For some immunofluorescence staining experiments (IFL), Caco2 cells were grown on eight-well slide culture chambers (Lab Tek II, Nunc, IL, USA) and were used 2 days after confluence.

### Bacteria strains and growth conditions

Wild-type *S*. Typhi strain Ty2 (Deng et al., 2003) was used in this study. Ty21a, a commercially available licensed live oral typhoid vaccine, is an attenuated strain developed by chemical mutagenesis of *S*. Typhi strain Ty2 (Germanier and Fuer, 1975). CVD 908-*htrA* and CVD 909 are Δ*aro* attenuated strains of *S*. Typhi (Tacket et al., 2000b, 2004). Bacteria were routinely pre-cultured at 37°C overnight in Luria-Bertani (LB) broth. Aliquots of the pre-cultures were inoculated into 5 ml DMEM and grown for 2–3 h at 37°C in a shaking incubator (200 rpm) until the cultures reached the exponential phase (OD_600nm_ = 0.5). The *aro* mutant strains were grown on *aro* agar, as previously described (Hone et al., 1991; Tacket et al., 2000b, 2004), pre-cultured in LB broth and cultured in DMEM with the addition of Dihydroxybenzoate (DHB, 0.1%).

### Generation of conditioned media and heat-killed cultures

Aliquots of overnight pre-cultured bacteria were grown in DMEM for 2–3 h to a final OD_600_ of 0.5. Cells were pelleted and supernatants filter sterilized by passing through a 0.22 μm pore size filter. Supernatants were used immediately upon filtration. These supernatants are hereafter referred to as “conditioned media” (CM).

Bacteria grown in DMEM for 2–3 h as described above were heat-killed (HK) by boiling at 100°C for 30 min. In both cases, the effectiveness of filtration and killing by heat was confirmed by lack of bacterial growth from 100 μl of these media plated onto agar plates and then incubated overnight at 37°C.

### Measurement of TEER

Trans-Epithelial Electric Resistance was used to monitor the integrity of the epithelial monolayer using a Millicel ERS Volt-ohm meter (World Precision Instruments, New Haven, CT, USA). Only those monolayers that exhibited a TEER of ∼1100–1700 Ω.cm^2^ were considered to have an appropriate barrier function and were used in the study. The average number of cells/monolayer was approximately about 1–1.5 × 10^5^ at confluency.

Cell monolayers were drained of media, gently washed with PBS and then incubated with DMEM without antibiotics and serum at 37°C for 2 h before bacterial infection.

The bacteria suspension, HK bacteria, or bacterial supernatants were added apically at an inoculation ratio [Multiplicity Of Infection (MOI)] of 40:1, 400:1, and 4000:1 bacteria:epithelial cell ratios, corresponding to 4–6 × 10^6^, 4–6 × 10^7^, and 4–6 × 10^8^ CFUs, respectively and incubated at 37°C. After 4 h infection, cells were washed with PBS to remove non-adherent bacteria and treated with gentamicin (480 μg/ml). Monolayers were incubated at 37°C overnight. TEER was measured at 2, 4, and 22 h post-infection.

### Cell viability

The viability of the Caco2 cells after bacterial infection with wild-type *S*. Typhi Ty2, Ty21a, CVD 908-*htrA*, and CVD 909, was assessed by a lactate dehydrogenase (LDH) secretion assay. The LDH secretion was measured from the cellular supernatant by a commercially available LDH assay kit (Cytotox 96, Promega, WI, USA) and carried as described by the manufacturer’s instructions. Lysis of the cells with 1% Triton X-100 served as positive control. The absorbance was measured at 490 nm using a multifunctional microplate reader. Cell viability was also assessed by Propidium Iodide staining by flow cytometry following a standard technique (Diebel et al., 2005; Riccardi and Nicoletti, 2006).

### Assessment of Caco2 cell monolayer paracellular permeability

The permeability of the Caco2 cell monolayers was evaluated by measuring the influx of Fluorescein isothiocyanate (FITC)-dextran and FITC-Bovine Serum Albumin (BSA) [molecular weights of 4.0 and 40 kDa (Sigma), respectively].

Fluorescein isothiocyanate-dextran and -Bovine Serum Albumin were dissolved in *P* buffer (10 mM HEPES, pH 7.4, 1 mM sodium pyruvate, 10 mM glucose, 3 mM CaCl_2_, 145 mM NaCl) or P/EGTA buffer [10 mM HEPES, pH 7.4, 1 mM sodium pyruvate, 10 mM glucose, 145 mM NaCl, 2 mM ethylene glycol-bis(ß-aminoethyl ether)-*N*,*N*,*N*′,*N*′-tetraacetic acid (EGTA)].

Briefly, the apical surface of Caco2 cell monolayers was infected with bacteria (MOI 400:1) for 4 h, washed, treated with gentamicin, and incubated at 37°C overnight. To measure the paracellular flux, the apical, and basolateral cell culture media were replaced with P buffer containing FITC-dextran (10 mg/ml) or FITC-BSA (10 mg/ml) and P buffer alone, respectively. P/EGTA buffer containing FITC-dextran (10 mg/ml) or FITC-BSA (10 mg/ml) and P/EGTA buffer were used as positive controls. After incubation for 4 h, the amounts of FITC-dextran and FITC-BSA in the basolateral media were measured with a fluorometer (excitation at 492 nm and emission at 520 nm). Data are expressed as fluorescent intensity.

### Cytokine assays

To determine the cytokine response of Caco2 cell monolayers to bacterial infection, cells were incubated with increasing amounts of bacteria (MOIs of 40:1, 400:1, and 4000:1 bacteria:cell ratios). After 4 h of incubation, bacteria were removed and monolayers incubated with fresh culture medium at 37°C overnight. After 22 h media were collected from either upper or lower chambers of each transwell. Samples were centrifuged at 2,000 rpm for 10 min to remove any residual cells or debris. Supernatants were stored at -80° and then assayed for interleukin (IL)-8, IL-6, tumor necrosis factor (TNF)-α, IL-17, IL-1β, interferon (IFN)-γ, and transforming growth factor (TGF)-β using a Luminex 100 platform (Luminex, Austin, TX, USA) and the Milliplex MAP High Sensitivity Human Cytokine kit. Kits were run according to the manufacturer’s instructions, with the exception of sample collection and processing as described above. Incubation of beads and test samples for all kits was performed overnight at 4°C for maximum sensitivity. Samples were run in duplicate. Media collected from Ty21a infected cells at a MOI of 400:1, were processed for detection of IL-8 and IL-6, only.

### Immunfluorescence

Monolayers on chamber slides (4 h post-infection staining) or transwell filter inserts (22 h post-infection staining) following infection were washed three times with PBS and fixed in PFA 4%/PBS for 20 min at room temperature. Cells were then blocked with 2% PBS-diluted normal goat serum (blocking solution) for 30 min and incubated with blocking solution-diluted primary antibody overnight at 4°C [anti zonula occludens (ZO)-1, 1:100].

After three washes with PBS, monolayers were incubated with TRITC-conjugated secondary antibody (1:5000 in blocking solution) at room temperature for 1 h in the dark. Phalloidin staining (1:1000) of actin filaments was carried out at the same time as the secondary antibody. Monolayers were washed with PBS and nuclei stained with DAPI (1:1000 in PBS) solution for 2 min at room temperature. Tissue culture filters housing the epithelial cell monolayers were carefully detached from their support and mounted on coverslips. Monolayers were analyzed with a Nikon Eclipse TE2000-E fluorescent microscope.

### Western blot analysis

#### Triton X-100-soluble and -insoluble fractions

Triton X-100-soluble and -insoluble fractions are working definitions applied to biochemically define the localization of tight junction proteins. Several studies have made use of this method to fractionate tight junctional protein in their cytosolic and membrane bound components (Youakim and Ahdieh, 1999; Nusrat et al., 2000, 2001; Andreeva et al., 2001; Chen et al., 2002). Proteins found in the Triton X-100-insoluble fraction have been associated with the tight junction complex.

Bacteria were added to the apical surface of Caco2 cell monolayers at a MOI of 400:1 for 4 h at 37°C. Bacteria were removed, cells were washed with PBS and incubated with DMEM supplemented with gentamicin. Cells were harvested at 4 and 22 h post-infection and Triton X-100-soluble and -insoluble protein fractions were prepared. Monolayers were harvested on ice in lysis buffer [1% Triton X-100, 100 mM NaCl, 10 mM HEPES, 2 mM EDTA, 4 mM Na3VO4, 40 mM NaF 200 mM PMSF, and a protease inhibitor cocktail (Complete Mini, Roche Molecular Biochemicals, Mannheim, Germany) and phosphatase inhibitors (Sigma, St. Louis, MO, USA)]. Lysates were rotated at 4°C, 30 min, centrifuged (14000 *g* for 30 min at 4°C) and the supernatant suspension, representing the Triton X-soluble fraction, was collected. The remaining pellet was re-suspended in lysis buffer supplemented with 1% SDS and sonicated (5W, 5 s) two–three times on ice. The resulting suspension was centrifuged (14000 *g* for 5 min at 4°C) and the supernatant, representing the Triton X-insoluble fraction, was collected. Samples were used immediately or stored at −80°C. Protein concentration was quantified by the Bradford method (Bio-Rad, Hercules, CA, USA). Samples were electrophoresed through a 10–20% gradient SDS polyacrylamide gel and transferred onto polyvinylidene difluoride membranes (Millipore, Bedford, MA, USA). Membranes were blocked in blocking buffer (Tris-buffered saline, 0.1% Tween 20, 5% BSA), for 1 h at room temperature. The blots were incubated overnight at 4°C with mouse anti-occludin diluted in blocking buffer. After washing, membranes were incubated for 1 h at room temperature with the appropriate secondary antibody diluted in blocking buffer. The hybridized band was detected by chemiluminescence using an ECL kit (Amersham) according to the manufacturer’s instructions. Membranes were stripped and reprobed (Blot restore solution, Millipore) for the detection of phosphothreonine followed by actin that served as loading control. Band intensity was normalized to actin and quantitated by densitometry using Image J software (National Institutes of Health). Data represent the average of two separate experiments.

### Antibodies

Mouse anti-occludin (OC-3F10, cat # 33–1500), mouse anti-ZO-1 (1A12, cat # 339100), and rabbit anti-phosphothreonine (ZPT1cat # 718200) antibodies were purchased from Invitrogen (Camarillo, CA, USA). Mouse anti-actin protein antibody (5C, cat # 82353) was purchased by Thermo Fisher Scientific (IL, USA). TRITC-conjugated anti-mouse and anti-rabbit secondary antibodies and FITC-conjugated phalloidin were obtained from Sigma (S. Louis, MO, USA).

### Statistics

Data are expressed as means ± SEM. Data were analyzed by using GraphPad (San Diego, CA, USA) software. Two-way (TEER) and one-way ANOVA were used to compare the data among groups. Differences were considered to be statistically significant if “*P*” values were <0.05.

## Results

### *Salmonella enterica* serovar Typhi affects epithelial barrier function by decreasing TEER in a dose-dependent fashion

**Figure 1:**
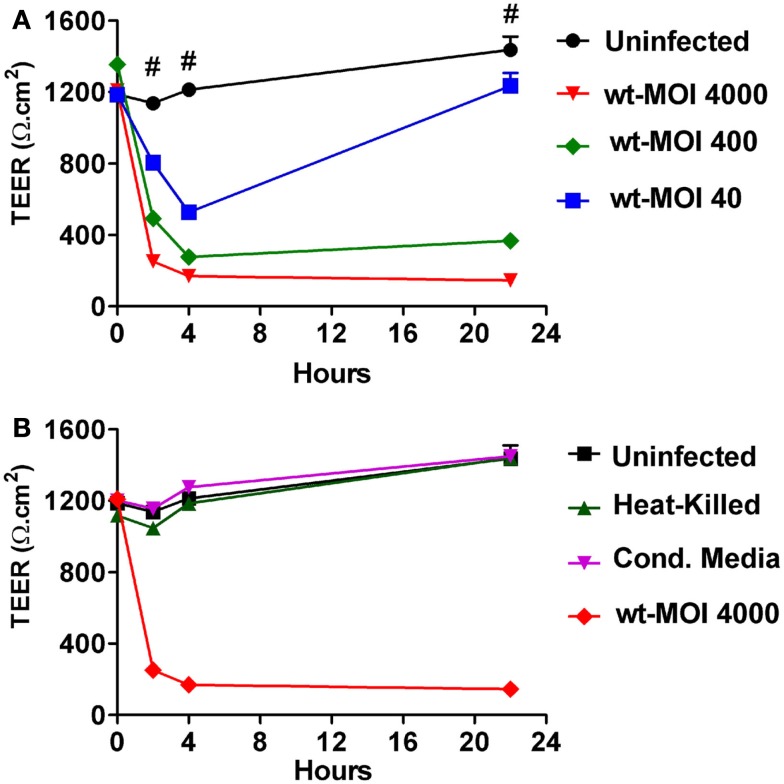
**Trans-epithelial electrical resistance (TEER) responses of CaCo2 cell monolayers to wild-type *S*. Typhi, HK bacteria, and culture supernatants**. **(A)** TEER changes upon infection with wild-type *S*. Typhi at different MOIs. **(B)** TEER in HK bacteria and culture supernatants (Cond. Media) treated monolayers. Data are expressed as means ± SEM for triplicate samples for all conditions tested. These results are representative of three experiments with similar results. #Denotes *p* < 0.001 of *S*. Typhi strains over *t* = 0 (ANOVA).

To investigate the effects of *S*. Typhi on mucosal barrier integrity, we infected Caco2 monolayers with *S*. Typhi at different MOIs. Since modulation and/or disruption of epithelial barrier function can be measured by changes in TEER, we used this technique to monitor alterations in mucosal permeability caused by the bacteria. As shown in Figure 1A we found that wild-type *S*. Typhi induced a decrease in TEER in the monolayer in a dose-dependent manner. All inoculation ratios used (MOIs of 40:1, 400:1, and 4000:1 bacteria:cell ratios, respectively) caused a significant drop in TEER [from 1147.0 ± 21.2 Ω.cm^2^ at baseline to 804.1 ± 37.5 Ω.cm^2^, 318.7 ± 38.2 Ω.cm^2^, and 252.6 ± 8.1 Ω.cm^2^, respectively] as early as 2 h post-infection. The decrease in TEER was even more dramatic at 4 h post-infection with TEER values of 527.6 ± 34.9 Ω.cm^2^, 206 ± 15.5 Ω.cm^2^, and 170.1 ± 1.6 Ω.cm^2^, respectively compared to baseline (1185.2 ± 40.0 Ω.cm^2^). At 4 h, bacteria were removed and monolayers were treated with gentamicin in order to eliminate non-adherent bacteria and incubated in medium overnight at 37°C. At 22 h post-infection, TEER values were still low for the monolayers infected at higher titers (298.8 ± 17.5 Ω.cm^2^ and 146.3 ± 7.4 Ω.cm^2^ for MOIs of 400:1 and 4000:1, respectively). In contrast, at a bacterial MOI of 40:1 we observed a substantial recovery in TEER, although still significantly lower than baseline (1235.3 ± 71.4 Ω.cm^2^ compared to a baseline value of 1487 ± 75.0 Ω.cm^2^) (Figure 1A). Taken together, these data demonstrate that *S*. Typhi alters Caco2 monolayer barrier function in a dose-dependent manner. Interestingly, the removal of bacteria allows the cells to slowly recover and counteract the adverse effect caused by the pathogen. This process apparently starts earlier for lower bacterial loads, presumably because the damage to the barrier function is less severe.

We then investigated whether the effects seen with wild-type *S*. Typhi on Caco2 cell TEER required the interaction of viable bacteria with the target enterocyte in order to elicit the host response. We thus compared wild-type with HK and filtered wild-type bacteria (CM). As shown in Figure 1B both the HK bacteria and the CM from *S*. Typhi, applied at the same titer as the wild-type strain, failed to significantly decrease the TEER of Caco2 cell monolayer. These results demonstrate that the effects of *S*. Typhi on barrier function depend on direct interaction of viable bacteria with target enterocytes and are not mediated by secretion of toxins or other mediators.

### Amelioration of epithelial barrier changes exhibited by attenuated *S*. Typhi vaccine strains

**Figure 2:**
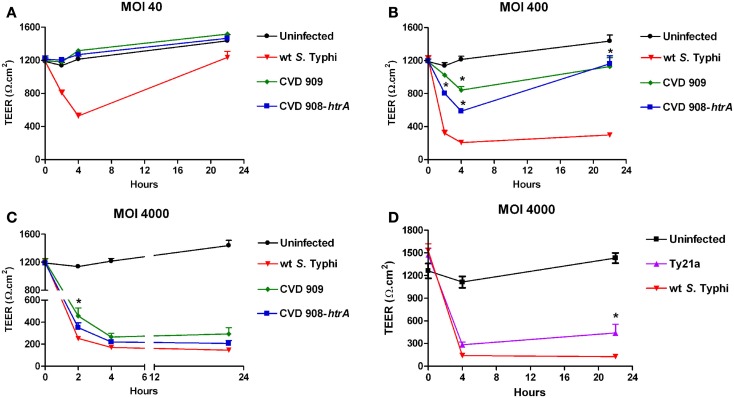
**The effect of *S*. Typhi attenuated strain on the TEER of polarized Caco2 monolayers**. Wild-type *S*. Typhi served as control. **(A)** *Aro* mutants-infected monolayers (MOI of 40:1); **(B)** *Aro* mutants-infected monolayers (MOI of 400:1). **(C)** *Aro* mutants-infected monolayers (MOI of 4000:1). **(D)** TEER in Caco2 cells infected with Ty21a applied apically at a MOI of 4000:1. Data are expressed as means ± SEM for triplicate samples for all conditions tested. These results are representative of three experiments with similar results. Statistical comparisons over wild-type *S*. Typhi at the same time point; *p < 0.05 (ANOVA).

To determine the effect of attenuated mutant strains of *S*. Typhi on mucosal permeability *in vitro*, Caco2 cell monolayers were infected with *S*. Typhi Ty21a, CVD 908-*htrA*, and CVD 909 attenuated strains (Figure 2). Wild-type *S*. Typhi was used as a positive control. Monolayers reached confluence in about 2 weeks, with a baseline TEER between 1200 and 1700 Ω.cm^2^ (*t* = 0). As described in our first series of experiments, wild-type *S*. Typhi induced a significant decline in TEER at all MOIs as early as 2 h post-infection (Figure 2). In contrast, both CVD 908-*htrA* and CVD 909 mutant strains failed to induce TEER changes at their lowest infection titers (MOI 40:1; Figure 2A). At a MOI of 400:1 (Figure 2B) we observed a decrease in TEER as early as 2 h post-infection that became more pronounced at 4 h when we registered a drop to 842 ± 43.5 Ω.cm^2^ and 586.75 ± 13.3 Ω.cm^2^ for CVD 909 and CVD 908-*htrA*, respectively compared to baseline values (1185.2 ± 40.0 Ω.cm^2^). Interestingly, 22 h after exposure to these attenuated strains we observed a recovery in TEER values, although the difference with the uninfected monolayer was still significant (Figure 2B).

Of note, at the highest bacterial load (MOI 4000:1) the resulting effects of the two *S*. Typhi vaccine candidates became similar to TEER decrease caused by the wild-type strain. At 22 h the TEER for all strains was very low, reaching its nadir at 293.5 ± 57.4 Ω.cm^2^ for the attenuated CVD 909 strain (Figure 2C). The only statistically significant difference we observed with the wild-type was for CVD 909 at 2 h post-infection. The vaccine strain Ty21a was tested at MOIs of 4000 and 400 in parallel with wild-type *S*. Typhi, as positive control. As shown in Figure 2D, at a MOI of 4000:1 Ty21a induced a drop in TEER (283.3 ± 37.1 Ω.cm^2^) similar to the wild-type (139.5 ± 5.3 Ω.cm^2^) at 4 h post-infection. At 22 h, we observed a slight recovery of TEER values for the mutant infected cells (440.1 ± 114.9 Ω.cm^2^) suggesting a less disruptive effect of this strain on Caco2 monolayer barrier function than wild-type *S*. Typhi (126.7 ± 5.1 Ω.cm^2^). At a bacterial infection load of 400:1 the effects of Ty21a on TEER were again similar to those observed with the wild-type strain with values at 22 h of 349.9 ± 56.3 Ω.cm^2^ (*p* = ns) compared to 194.7 ± 15.3 Ω.cm^2^ with wild-type *Salmonella* (data not shown).

These data demonstrate that the new generation mutant strains tested, although capable of affecting the integrity of the epithelial cell monolayers at high inoculation ratios, have a considerably milder effect on the intestinal barrier integrity than wild-type *S*. Typhi at lower inoculation ratios. Ty21a effect on Caco2 cell TEER was overall similar to that of wild-type *Salmonella*.

### Effects of *S*. Typhi infection on cellular viability

**Figure 3:**
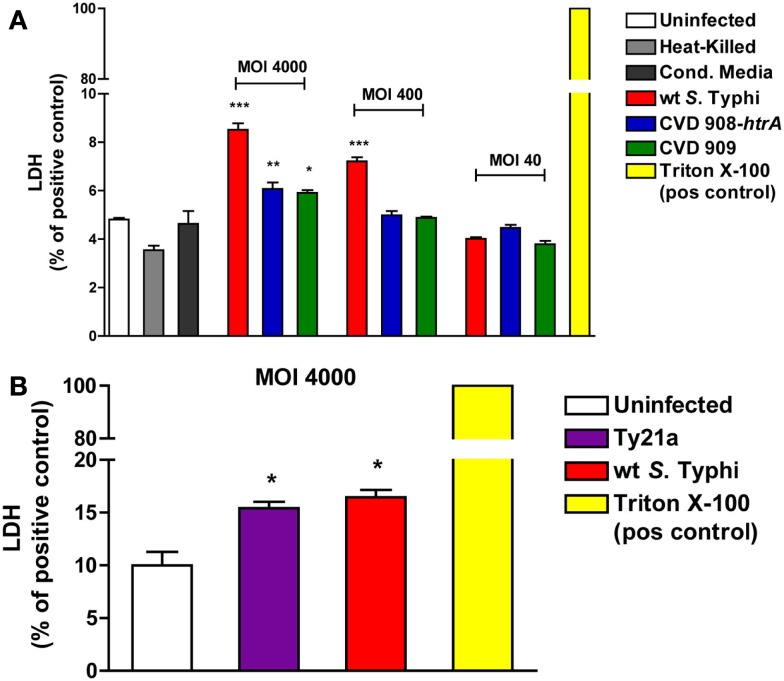
**Effect of *S*. Typhi infection on cell viability**. LDH levels were measured in the apical media as an index of cell death. Values represent LDH release as a percentage of total LDH (positive control, Triton X-100). **(A)** LDH levels after infection with wild-type *S*. Typhi and its *aro* mutants at different MOI’s. **(B)** LDH release after infection with wild-type *S*. Typhi and Ty21a applied at a MOI of 4000:1. Data are expressed as means ± SEM for triplicate samples for all conditions tested. These results are representative of three experiments with similar results. ***p < 0.001, **p < 0.01, *p < 0.05 compared to uninfected cells (ANOVA).

In order to evaluate if the disruption of the epithelial barrier function caused by *S*. Typhi is mediated by enhanced cell death, the viability of the monolayer was assessed by measuring the levels of LDH released in the cell medium at 22 h post-infection. As shown in Figure 3A, all strains induced a significantly higher release of LDH compared to uninfected cells, at the highest MOI (4000:1). At a MOI of 400:1 only wild-type *S*. Typhi induced a significant higher release of LDH. No differences with the uninfected cells were observed at a MOI of 40:1 for any of the strains studied. Similarly, as shown in Figure 3B, a significantly higher level of LDH was observed only for the wild-type Ty2 *S*. Typhi at a MOI of 4000:1 compared to uninfected controls. These results were confirmed by staining with Propidium Iodide and flow cytometry (data not shown). The range of dead cells varied between 4.3% in the uninfected monolayers and 13.7% in cultures with wild-type *S*. Typhi at a MOI of 4000.

These data show that, although all strains can affect cell viability when applied at high doses, the overall monolayer viability is mostly preserved, with the greatest cytotoxicity level observed being less than 20% of the positive control. Taken together with the data discussed above showing that at a bacterial load of 40:1 there is a recovery of TEER over time and even at a MOI of 400:1 we observed the complete recovery of TEER after 3 days (data not shown), these data suggest that the decrease observed is likely to be largely due to modulation of cellular permeability, with only a minor component attributable to cell loss or toxicity resulting in cell death.

### *S*. Typhi infection increases paracellular flux in Caco2 cell monolayers

**Figure 4:**
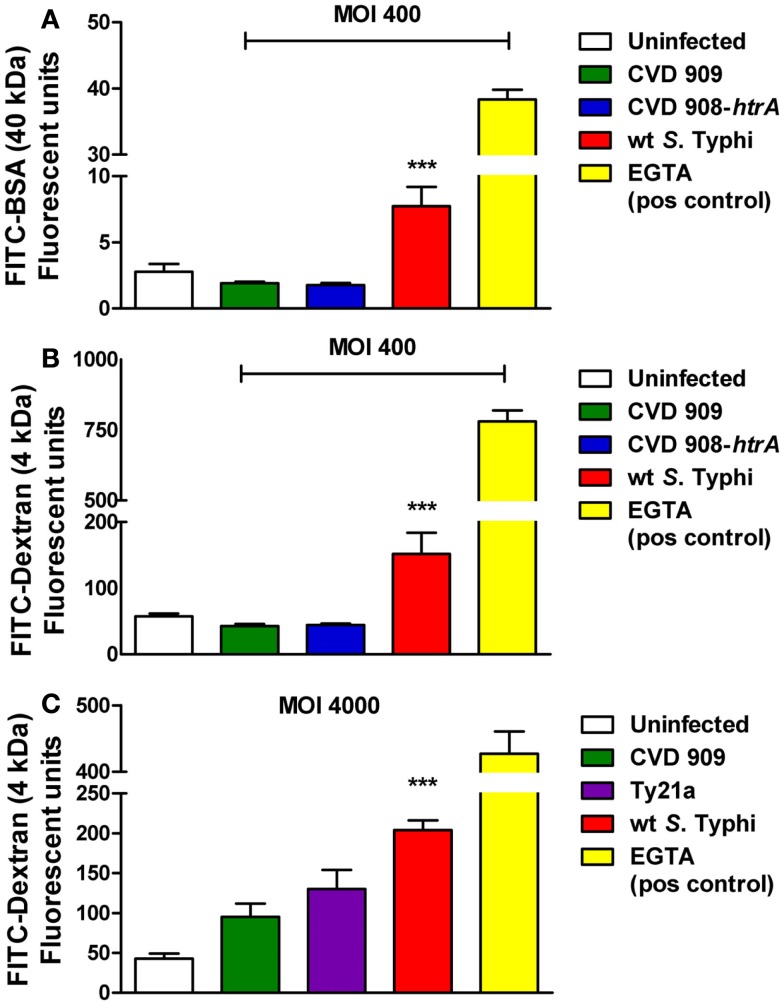
**Paracellular permeability in Caco2 cell monolayers after infection with wild-type *S*. Typhi and its mutant strains**. **(A)** FITC-BSA (40 kDa) net transport after infection with wild-type *S*. Typhi at a MOI of 400:1. **(B)** FITC-Dextran (4 kDa) net transport after infection with wild-type *S*. Typhi or the attenuated *aro* mutants CVD 908-*htrA* and CVD 909, applied at a MOI of 400:1. **(C)** Permeability to FITC-Dextran (4 kDa) after infection with wild-type *S*. Typhi and the vaccine strain Ty21a at a MOI of 4000:1. Calcium-free medium supplemented with EGTA to disrupt TJs served as positive control. Results are expressed as mean ± SEM of triplicate samples for each condition and are representative of three experiments with similar results. ***p < 0.001 compared to uninfected cultures (ANOVA).

**Figure 5:**
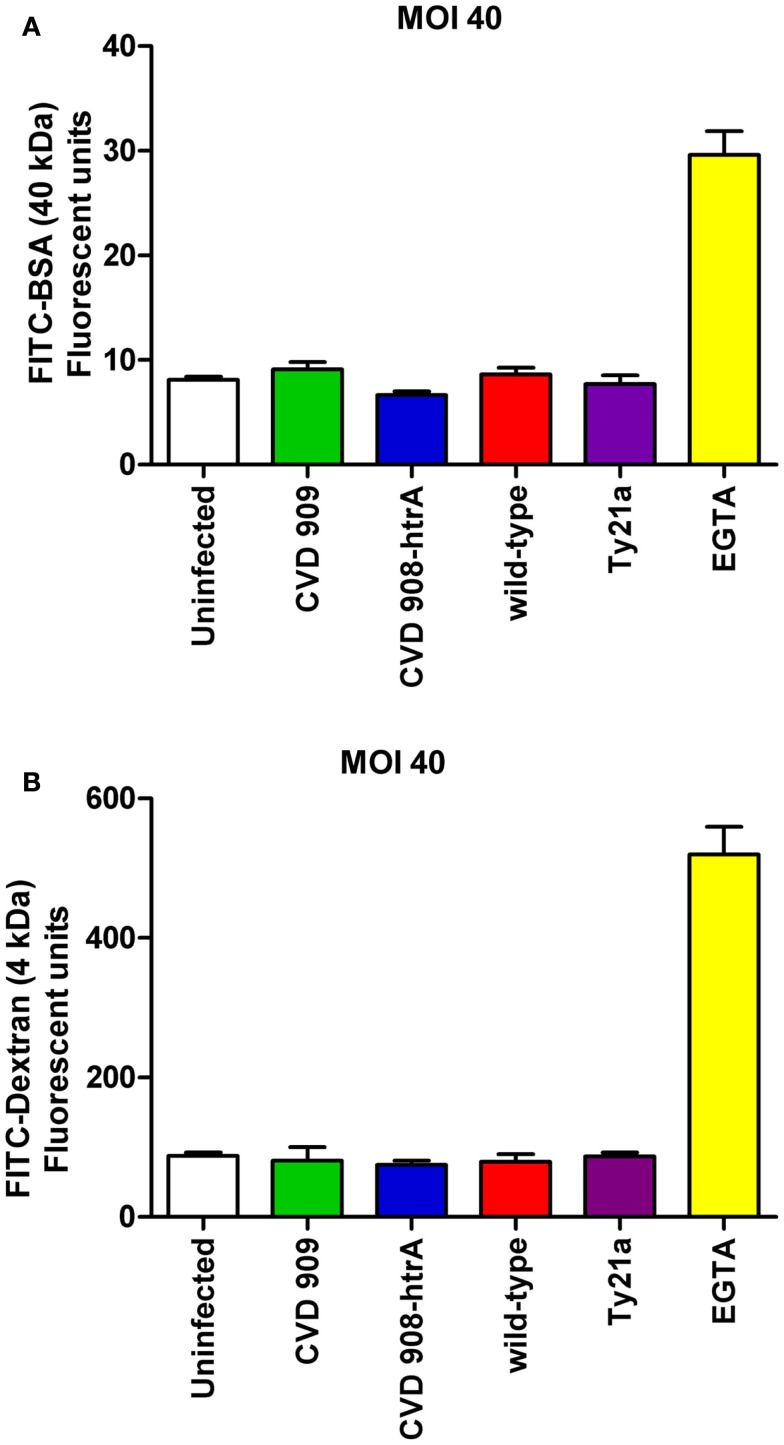
**Paracellular permeability in Caco2 cell monolayers after infection with wild-type *S*. Typhi and its mutant strains (MOI 40:1)**. **(A)** FITC-BSA (40 kDa) net transport after infection with wild-type *S*. Typhi and attenuated strains. **(B)** FITC-Dextran (4 kDa) net transport after infection with wild-type *S*. Typhi or the attenuated mutants. Calcium-free medium supplemented with EGTA to disrupt TJs served as positive control. Results are expressed as mean ± SEM of triplicate samples for each condition and are representative of three experiments with similar results. *p* = ns compared to uninfected controls (ANOVA).

We next sought to determine whether alterations induced by *S*. Typhi with regard to TEER correlated with changes in epithelial paracellular permeability. We evaluated alterations in barrier function in response to the pathogen, by measuring the trans-epithelial flux of fluorescently labeled Dextran (FITC-dextran, 4 kDa) and BSA (FITC-BSA, 40 kDa). As shown in Figure 4, Caco2 cells exposed to wild-type *S*. Typhi showed a significantly increased transport of both FITC-BSA (Figure 4A) and FITC-Dextran (Figures 4B,C) from the apical chamber to the basolateral side compared to uninfected monolayers. None of the mutant strains caused increased paracellular permeability to these markers. (Figures 4A–C). At a MOI 40:1, wild-type *S*. Typhi failed to increase the paracellular flux of labeled markers (Figure 5). EGTA was used as a maximum permeability positive control for its ability to completely open tight junctions. As expected, EGTA-treated monolayers showed a remarkably higher degree of transport of both tracers confirming the validity of the assays. Although we can’t exclude that to some extent the paracellular flux of labeled markers might be due to the minimal cell death we observed when infecting monolayers with wild-type *S*. Typhi, these results likely represent a *S*. Typhi-mediated increase in epithelial permeability resulting from modulation of intercellular tight junctions function. In light of this, the mutant vaccine strains appear to have either an attenuated or no effect on the tight junction barrier.

### *S*. Typhi affects paracellular permeability by altering tight-junction proteins distribution and scaffolding in polarized cellular monolayers

We have determined that *S*. Typhi affects epithelial barrier integrity largely by increasing Caco2 monolayer paracellular permeability. The maintenance of TEER and the relative impermeability of polarized epithelium to macromolecules require the correct functioning and integrity of intercellular tight junctions and their association with cytoskeletal proteins at the apico-lateral cell surface (Balda and Matter, 1998). In order to investigate the possible disruption of the tight junctional complex by the pathogen, we examined the tight junction-associated protein ZO-1 in infected monolayers by immunofluorescence microscopy.

**Figure 6:**
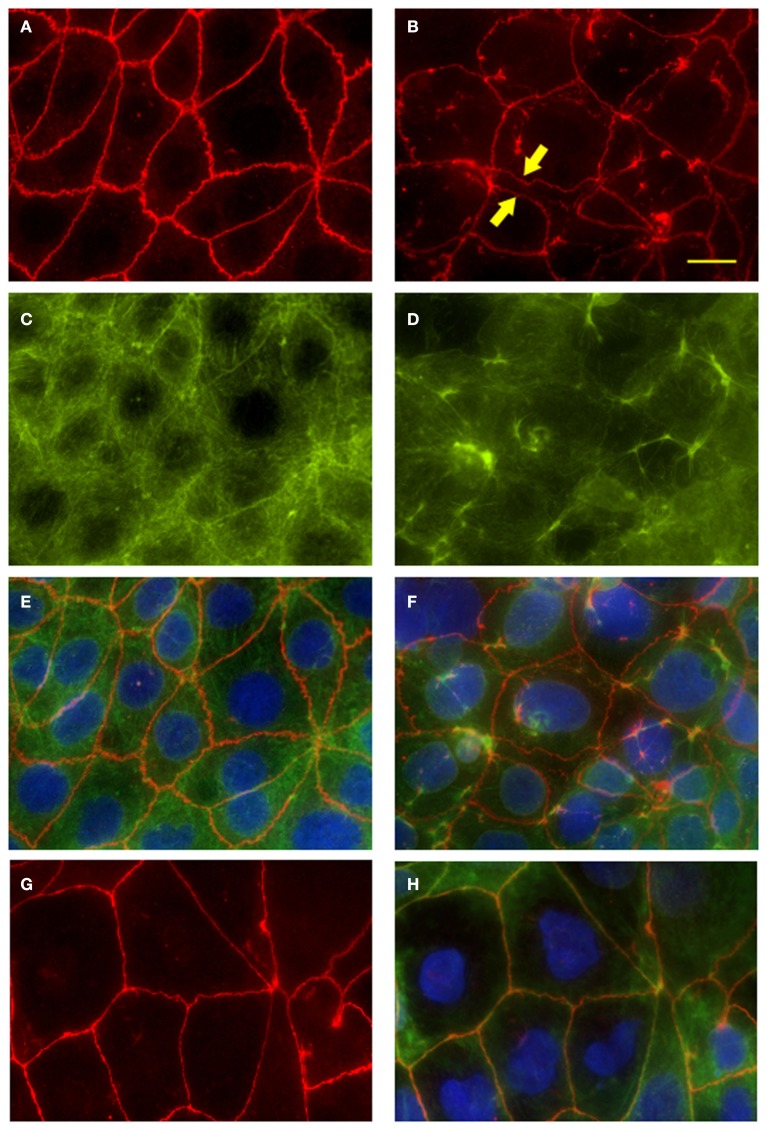
**Fluorescence microscopy of Caco2 monolayers labeled with ZO-1 or fluorescein-phalloidin (actin) show disruption of the tight-junction complex and the cytoskeleton at 4 h post-infection**. Caco2 polarized monolayers were infected with wild-type *S*. Typhi (MOI 400:1) for 4 h, washed with PBS, fixed and stained along with uninfected controls. **(A)** ZO-1 staining in uninfected monolayers. Note the characteristic chicken wire patterning. **(B)** Disrupted ZO-1organization in wild-type *S*. Typhi -infected monolayers. Areas of cell-cell detachment are marked by arrows. **(C)** Actin staining of the cytoskeleton in uninfected controls. **(D)** Actin fibers stained in the *S*. Typhi -infected cells. **(E)** Merge of **(A,C)**. **(F)** Merge of **(B,D)**. **(G)** ZO-1 distribution in CVD 909 infected monolayers. **(H)** ZO-1 (red), actin (green) and DAPI for the nuclei (blu) merged staining of CVD 909 infected cells. Bar, 25 μm.

**Figure 7:**
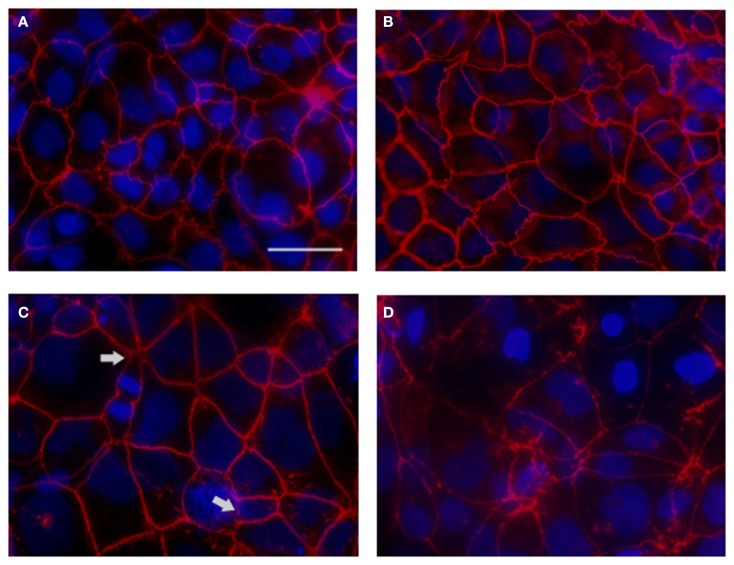
**ZO-1 staining of *S*. Typhi and mutant strains infected monolayers at 22 h post-infection**. **(A)** Uninfected control. **(B)** CVD 909. **(C)** Ty21a. **(D)** Wild-type *S*. Typhi. ZO-1 staining (red), DAPI for the nuclei (blu). Areas of severe ZO-1 disruption are marked by arrows. Bar, 30 μm.

As shown in Figure 6A, in uninfected monolayers ZO-1 is localized at the cell-cell boundary in a typical chicken wire-like pattern throughout the monolayer, indicating intact tight-junction complexes. In contrast, following infection with wild-type *S*. Typhi for 4 h (Figure 6B; MOI 400:1), ZO-1 distribution appears altered: while still observing the protein at the cell boundaries, ZO-1 also appeared clustered into the cytoplasm and co-localized with aggregates of actin fibers of the disrupted cytoskeleton (Figures 6D,F). In contrast, as expected, in uninfected monolayers the actin cytoskeleton was organized in a network of filaments normally distributed beneath the plasma membrane and throughout the cytoplasm (Figures 6C,E). As a plausible effect of this tight-junction and cytoskeletal rearrangement we observed the detachment of adjacent cells from each other (Figure 6B, arrows). Similar to ZO-1, in infected monolayers we observed the clustering of claudin-1into the cytoplasm around the nucleus and the detachment of adjacent cells (data not shown). Consistent with TEER data, in monolayers infected with CVD 909 we did not observe major alterations of ZO-1 localization. As shown in Figures 6G,H, ZO-1 is localized at the cell-cell boundary with minimal internalization and also minimal alteration of the actin cytoskeleton. At 22 h post-infection, we still observed a severely altered distribution of ZO-1 in wild-type *S*. Typhi treated cells (Figure 7D) whereas the effect appears largely attenuated in Ty21a infected cells, for which only a few areas of cell–cell detachment and some ZO-1 internalization were detected (Figure 7C, arrows). No effect on the distribution of ZO-1 was observed with CVD 909 (Figure 7B) or CVD 908 (not shown). These results strongly support the notion that changes in epithelial permeability induced by bacterial infection are caused by alterations in the paracellular pathway due to disruption and/or modulation of the sealing function of tight junctions.

### Phosphorylation of occludin plays a role in the regulation of tight junction function upon *S*. Typhi infection

Occludin has been widely described to play an important role in the regulation of tight-junction integrity (Rao, 2009). It has been shown that its overexpression results in TEER elevation (Balda et al., 1996; McCarthy et al., 1996). We have analyzed the solubility of occludin in the non-ionic detergent Triton X-100 as an indicator of its association with the tight-junction complex, in uninfected and infected monolayers in order to determine its implication in *S*. Typhi induced increased permeability of Caco2 monolayers.

**Figure 8:**
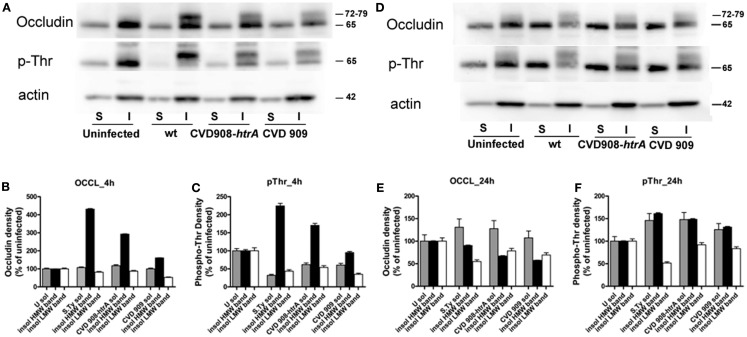
**Occludin is hyperphosphorylated on threonine and translocates into the cytoplasm**. We analyzed the solubility of occludin as an indication of its association with tight junctions. Protein lysates in the form of X-100 Triton soluble (S) and insoluble (I) fractions were obtained from Caco2 cell monolayers after infection with wild-type *S*. Typhi, CVD 908-*htrA*, and CVD 909 for 4 and 22 h. Equal amounts were loaded on the gel, electrophoresed, transferred onto a membrane and blotted with antibodies. **(A)** Western blot of protein samples blotted with anti-occludin (upper panel), anti-phosphothreonine (middle panel), and anti-actin (lower panel) as loading control, 4 h following exposure to bacteria. **(B,C)** Quantification of the data shown in **(A)**. **(D)** Western blot of protein samples blotted with anti-occludin (upper panel), anti-phosphothreonine (middle panel) and anti-actin (lower panel) 22 h post-infection with *S*. Typhi strains. **(E,F)**. Quantification of the data shown in **(D)**. Data have been normalized to the actin loading control. These results represent the average of two individual experiments.

After infection, equal protein amounts of cell lysates from the Triton X-100 soluble and insoluble fractions were resolved by SDS-PAGE and then analyzed by immunoblotting in parallel with uninfected controls (Figure 8). In the uninfected monolayer occludin was mainly localized in the insoluble fraction and is visible on western blots as a strong band of about 65 kDa [Low Molecular Weight (LMW); Figure 8A, upper panel]. Upon infection with wild-type *S*. Typhi, an additional band of about 72–79 kDa was detected in the insoluble membrane fraction at 4 h post-infection. This High Molecular Weight (HMW) band was also observed in both CVD mutants-treated cell lysates, although weaker than that observed with the wild-type *S*. Typhi (Figure 8A, upper panel; Figure 8B). At 22 h, we observed an increase in LMW occludin in the soluble fraction paralleled by a decrease of the HMW species in all bacteria samples (Figure 8D, upper panel; Figure 8E). The HMW species (72–79 kDa) has been previously shown to represent a hyperphosphorylated form of occludin and represents a sub-pool of this protein specifically associated with the functional sealing components of tight-junction (Sakakibara et al., 1997; Wong, 1997; Seth et al., 2007). Specifically it has been demonstrated that occludin undergoes dephosphorylation on Ser/Thr residues during the disruption of tight junctions by various insults. Analysis of the threonine phosphorylation status of our samples showed that occludin appears to be phosphorylated on Thr in the resting epithelium (uninfected controls, LMW band, Figures 8A,D, middle panels). Conversely, in 4 h wild-type *S*. Typhi-infected cell lysates, only the HMW band appeared phosphorylated and this represents the hyperphosphorylated form of occludin. We observed a similar, albeit milder, shift of the occludin phosphorylation status toward the HMW species in both CVD 909 and CVD 908-*htrA* *S*. Typhi mutants (Figure 8C). Analysis of lysates at 22 h post-infection revealed that *S*. Typhi induced both a loss of occludin and hyperphosphorylated occludin from the insoluble fraction and a shift of the signal to the soluble fraction (Figure 8D, middle panel; Figure 8F) in all *Salmonella* strain-treated samples. As in the uninfected control, the phosphorylation status of the 65 kDa band of the insoluble membrane fraction was apparently not affected.

Taken together, these data clearly show that wild-type *S*. Typhi induces changes in the phosphorylation status of occludin and this is likely to result in the disruption of tight junctions.

### *S*. Typhi interaction with epithelial cells triggers the release of pro-inflammatory cytokines

Cytokines play a central role in regulating immune and inflammatory responses during infection with pathogens. Pro-inflammatory cytokines are crucial components of the host response to pathogenic microbes; they are produced early in infection and contribute to various steps of the host inflammatory and immune response. A few studies have shown that *S*. Typhi induces the release of IL-8 and/or IL-6 in epithelial cells (Weinstein et al., 1997, 1998; Sharma and Qadri, 2004; Raffatellu et al., 2005; Winter et al., 2008) however, no studies to date have evaluated the immune response elicited by the interaction of attenuated strains of *S*. Typhi with the host epithelial cells. Thus, we analyzed the early immune response of intestinal epithelial cells to infection with the *aro* mutants CVD 908-*htrA* and CVD 909 and the licensed vaccine Ty21a.

**Figure 9:**
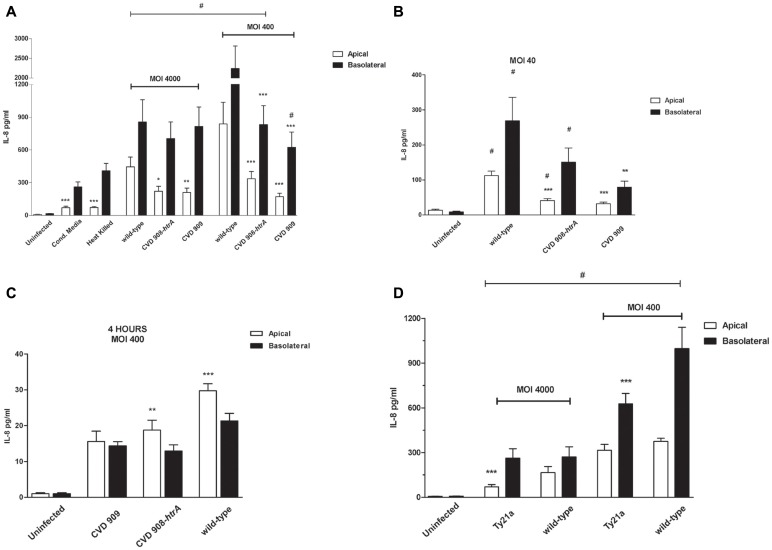
***S*. Typhi attenuated strains induce IL-8 secretion by Caco2 cells**. **(A)** IL-8 secreted by Caco2 cells infected with vaccine candidates CVD 908-*htrA* and CVD 909 applied at MOIs of 4000:1 and 400:1, HK *S*. Typhi and conditioned media (22 h post-infection). **(B)** IL-8 released by Caco2 cells infected with the *aro* mutants at a MOI of 40:1at 22 h post-infection; **(C)** IL-8 secretion measured at 4 h post-infection at a MOI of 400:1 (statistical analysis between the apical and basolateral compartments for the same strain); **(D)** IL-8 measured at 22 h after infection with the vaccine strain Ty21a applied at MOIs of 4000 and 400. Wild-type *S*. Typhi served as positive control. Values shown represent the mean ± SEM of three independent assays. #*p* < 0.05 over uninfected; ***p < 0.001, **p < 0.01, *p < 0.05 for comparisons between wild-type *S*. Typhi and mutant strains applied at the same titer (ANOVA).

Supernatants from both the monolayer apical and basolateral compartments were evaluated by Luminex assay for the detection of the pro-inflammatory cytokines IL-1β, IL-8, IL-6, TNF-α, IL-17, and IFN-γ. Moreover, we assayed supernatants for the measurement of IL-10 and TGF-β. Media collected from Ty21a infected cells were assessed for levels of IL-8 and IL-6 only. Cytokines were measured at 4 and 22 h post-infection. The only cytokines detected in these studies were IL-8 and IL-6. Levels of all other cytokines were very low or below detection. Figure 9A shows the results for IL-8 in media collected from the apical and basolateral sides of uninfected, live wild-type *S*. Typhi as positive control, filtrate, heat-inactivated *S*. Typhi, and *S*. Typhi *aro* mutants-infected Caco2 monolayers at 22 h post-infection. As expected, we detected a significant cytokine secretion in the basolateral compartment but interestingly a remarkable release of IL-8 was also measured on the apical side. IL-8 secretion induced by wild-type *S*. Typhi was significantly higher than that observed in uninfected cells on both sides and at all bacterial loads applied except for CVD909, apical secretion (Figure 9A). The largest IL-8 secretion was measured at a MOI of 400:1 for which we detected amounts of 2239 ± 573 and 838 ± 197 pg/ml compared to the uninfected monolayer, on the basolateral and apical sides, respectively. Even at the lowest MOI of 40:1 the fold increase was highly significant compared to uninfected, both on the basolateral (918 ± 163) and apical (340 ± 585; Figure 9B) side. IL-8 secretion in the basolateral side induced by filtered and heat-inactivated wild-type bacteria (Figure 9A) is remarkably lower than live, wild-type *S*. Typhi, suggesting that a physical interaction with the live pathogen is needed to trigger a strong cytokine response. *S*. Typhi *aro* mutants elicit remarkable levels of cytokine secretion, although the overall IL-8 amounts are lower than those secreted by wild-type bacteria-infected cells, in both the apical and basolateral compartments (Figures 9A,B). Similar to the wild-type strain, we measured the highest secretion of IL-8 at a MOI of 400:1 CFU/cell for CVD 908-*htrA*: 336.5 ± 66.5 and 832 ± 175 pg/ml in the apical and basolateral sides, respectively. IL-8 secretion elicited by the mutant strain CVD 909 was higher than untreated cells at all MOIs, with the greatest difference being observed at the MOI of 4000:1, with IL-8 levels 210 ± 40.1 and 814 ± 179 pg/ml compared to 7.49 ± 2.03 and 15.0 ± 2.94 pg/ml of uninfected cells, in the apical and basolateral compartments, respectively.

Although wild-type *S*. Typhi induces more IL-8 secretion than the mutant strains added at the same titers, data show that the differences are consistently statistically significant for the cytokine release in the apical compartment (Figures 9A,B). IL-8 amounts in the basolateral side were significantly different from wild-type strain only for both CVD 908-*htrA* and CVD 909-treated monolayers at the MOIs of 400:1 and for CVD 909 at 40:1. Between the two *aro* mutants, data show no significant differences at any concentration. We observed a slight although not significant increase in IL-8 secretion upon treatment of cells with filtered and heat-inactivated bacteria, compared to uninfected monolayer (Figure 9A). Interestingly, IL-8 levels measured at 4 h post-infection, were significantly higher in the apical side than the basolateral for both wild-type *S*. Typhi and CVD 908-*htrA* applied at a MOI of 400:1 (Figure 9C).

**Figure 10:**
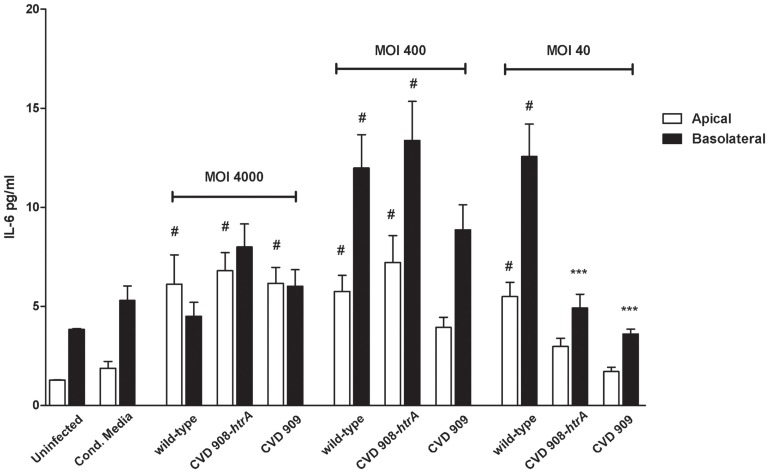
***S*. Typhi attenuated strains induce secretion of IL-6**. IL-6 secreted by Caco2 cells infected with vaccine candidates CVD 908-*htrA* and CVD 909 applied at different bacterial loads, conditioned media, and the wild-type strain, as positive control. Values shown represent the mean ± SEM of three independent assays. #*p* < 0.05 over uninfected; ***p < 0.001, **p < 0.01, *p < 0.05 for comparisons between wild-type *S*. Typhi and mutant strains applied at the same titer (ANOVA).

IL-8 secreted by Caco2 monolayers upon infection with Ty21a strain (MOI 400:1), was ∼50 and 100-fold greater than the uninfected control in the apical and basolateral sides, respectively (Figure 9D). Ty21a elicited a reduced IL-8 release compared to wild-type *S*. Typhi, with a significant difference in the basal compartment. At a higher MOI, significant difference was observed between wild-type and Ty21a only for IL-8 amounts released apically. Of note, in our *in vitro* system we did not observe any substantial difference in the ability of Ty21a to elicit IL-8 secretion when Ty21a was grown in the absence or presence of 0.05% galactose, a concentration which has been previously shown to allow complete LPS O-antigen synthesis without reducing the strain growth rate (Shi et al., 2010). As shown in Figure 10, IL-6 was secreted by all strains and at all conditions applied. IL-6 levels measured apically were significantly higher than uninfected cells for most of the strains and conditions except CM, CVD 908-*htrA* at a MOI of 40:1 and CVD 909 at MOIs of 40:1 and 400:1. On the basolateral surface IL-6 secretion levels appeared to be statistically higher than uninfected controls for wild-type *S*. Typhi and CVD 908-*htrA* applied at a MOI of 400:1 and *S*. Typhi applied at a MOI of 40:1. At the highest bacterial loads (MOIs 4000:1 and 400:1) we did not observe any significant difference in the level of IL-6 secretion between the wild-type strain and either vaccine candidate in both compartments; conversely, both CVD 908-*htrA* and CVD 909 applied at a MOI of 40:1 induced a basolateral secretion of IL-6 significantly lower than wild-type *Salmonella*. No statistical differences were observed between CVD 908-*htrA* and CVD 909 strains.

Taken together, these data suggest that *S*. Typhi induces a significant immune response by epithelial cells through the release of the pro-inflammatory cytokines IL-8 and IL-6. Of importance, these results provide additional information regarding the attenuation of the *S*. Typhi mutant strains CVD 908-*htrA*, CVD 909 and Ty21a as evidenced by their induction of measurable, but reduced responses compared to the wild-type strain, particularly at lower titers.

## Discussion

Bacterial enteric infections exact a heavy toll on human population, particularly among children. Despite the explosion of knowledge on the pathogenesis of enteric diseases experienced during the past decade, the number of diarrheal episodes and human deaths reported worldwide remains remarkably high. Typhoid fever is a serious, life-threatening disease widespread in developing countries, affecting about 21.5 million persons each year. About 400 individuals in the United States each year, 75% of which get infected during international travels develop typhoid fever. In addition to risk of death or persistent infection, other potential complications of *S*. Typhi infection include toxemia, myocarditis, liver damage and intestinal lesions. Due to increased resistance to antibiotics, the development of effective and safe vaccines against *S*. Typhi infection is a public health priority. The scientific effort to study the cross talk between enteric pathogens and intestinal host has been mainly focused on studying bacterial pathogenesis and how microorganisms can cause diarrhea. In this manuscript, we believe for the first time, we have shifted our attention from how bacteria can induce diarrhea and inflammation to the integrated response of the intestinal epithelial milieu following exposure to *S*. Typhi.

The mammalian gastrointestinal tract is lined by a dynamic epithelium exquisitely responsive to stimuli of innumerable variety, and is populated by a complex community of microbial partners, far more numerous than the cells of the intestine itself. In normal homeostasis, the GI epithelial layer forms a tight, but selective barrier: microbes and most antigens are held at bay, but nutrients from the essential to the trivial are absorbed efficiently. This selectivity is based on the capability of intestinal epithelial cells to perceive the luminal presence of possible “danger signals.” The interaction between the epithelium and the luminal microbial population is mainly based on modulation of intestinal permeability and intestinal mucosal defenses. The tightness of the epithelial barrier is itself dynamic, though the mechanisms governing and effecting dynamic permeability are only partially understood.

Our data demonstrate that exposure to *S*. Typhi causes a time- and dose-dependent impairment of the gut epithelial barrier function without overt damage of the intestinal monolayer. The doses of bacteria we used in this study range from 4 to 600 times lower than a single dose of the Ty21a typhoid vaccine. Although these bacterial amounts applied to the single monolayer on a membrane insert area of 1.1 cm^2^ might appear too high compared to a single dose of the vaccine, we have to consider that physiologically in the intestinal tract the distribution of the ingested vaccine won’t be homogeneous. There are sections of the GI tract, including the duodenum and the jejunum that will be exposed to a higher bacterial load than the ileum. Moreover, the numbers of bacteria present in particular GI segments are likely to be affected by many factors, including the preference of *S*. Typhi to colonize the small intestine, bacterial replication in the local microenvironment, numbers of typhoid bacilli present in acute or chronic infection, etc. Our study shows that at a lower *S*. Typhi MOI (40:1), Caco2 monolayers have an almost complete barrier function recovery (Figure 1A), while at higher concentrations there is a slight (MOI 400:1) or absent (MOI 4000:1) recovery of monolayer’s tightness at 22 h post-infection. After removing the bacteria, the monolayer starts slowly to recover and counteract the adverse effect caused by the pathogen. This process apparently begins earlier for lower bacterial loads, presumably because the damage to the barrier function is less severe. We have shown that although occurring, cell death is low and limited mostly to the higher bacteria infection ratios. Based on the results presented, we believe that cell death accounts only marginally for the increased permeability. In support of this, we were able to provide the molecular bases for the changes in barrier integrity by showing that intercellular tight junctions of Caco2 monolayers exposed to wild-type *S*. Typhi were disassembled (Figures 6–8) and by demonstrating that TJ disassembly was due to *S*. Typhi induced early (4 h) hyperphosphorylated occludin (Figures 8A–C), followed by a late (22 h) shift of the occludin signal to the soluble fraction (Figures 8D–F). These changes in occludin phosphorylation and localization were paralleled by similar departure of the scaffold proteins ZO-1 (Figures 6B and 7D) and claudin-1 (not shown) from cell boundaries. These data suggest that exposure of epithelial cells to *S*. Typhi causes phosphorylation of the transmembrane TJ component occludin, followed by translocation of occludin, ZO-1 and claudin-1 from cell boundary to cytoplasm, leading to increased paracellular permeability as demonstrated by the increased passage of both paracellular markers dextran and BSA from the apical to the basolateral monolayer compartments. Interestingly, *S*. Typhi vaccine candidates CVD 908-*htrA* and CVD 909 caused either no changes in TEER at lower doses (MOI 40:1) or less dramatic changes at higher doses compared to wild-type *Salmonella* (see Figure 2), paralleled by a decreased passage of paracellular markers dextran and BSA (Figures 4 and 5). Also it was interesting to note that the licensed and currently clinically used *S*. Typhi Ty21a typhoid vaccine caused TEER changes similar, although not as severe, as those observed with wild-type *S*. Typhi (Figure 2D). This was further supported by immunofluorescence staining of ZO-1 at 22 h, showing an attenuated effect on tight-junction barrier compared to the wild-type strain, although more severe than the CVD mutants (Figure 7C).

Ty21a is an attenuated mutant strain of *S*. Typhi Ty2, isolated in the early 1970s by random chemical mutagenesis. It has a prominent GalE- and Vi-negative phenotype (Germanier and Fuer, 1975) that, associated with further spontaneous mutations (Germanier and Fuer, 1975; Coynault et al., 1996), resulted in a vaccine exhibiting remarkable safety and good efficacy (Levine et al., 1999). The Ty21a is the active constituent of Vivotif^®^ (Berna Biotech Ltd., Switzerland), currently the only licensed live oral vaccine against typhoid fever. Because of the multiple doses needed to achieve protection, novel attenuated *S*. Typhi strains that may serve as single dose oral attenuated vaccines have been developed. CVD 908-*htrA* and CVD 909 are mutant strains carrying mutations in the *aro*matic amino acid synthesis pathway. In particular, deletions in the *aro* genes (Ty2 *aroC* and *aroD*) make these strains auxotrophic for some *aro*matic compounds. The mutant bacteria become attenuated because they are unable to scavenge these molecules in mammalian cells. A further deletion of the *htrA* gene impairs their response to stress and the ability to survive inside macrophages (Lowe et al., 1999). CVD 909 constitutively expresses the Vi antigen. Its invasiveness might be reduced compared to its isogenic parent, CVD 908-*htrA* and the wild-type since it has been shown *in vitro* that the expression of the Vi capsule is negatively associated with the expression of the genes regulating invasion such as flagellin and T3SS-1 (Arricau et al., 1998). It has been shown that Vi expression in *S*. Typhi is repressed in high osmolarity environments (such as the intestinal lumen) and viceversa is induced in conditions of low osmolarity [i.e., blood and tissue (Arricau et al., 1998)]. We have grown all our strains in LB and/or DMEM media, mimicking the physiological environment of the intestinal lumen and our invasion assays confirm that CVD 909 is less invasive than its isogenic mutant CVD 908-*htrA* (data not shown), most likely due to the constitutive expression of the Vi antigen. While to a lesser extent than CVD 909, the CVD 908-*htrA* and Ty21a mutants showed a less invasive phenotype as compared to the wild-type, as well (data not shown), reflecting their overall attenuated properties. Although it might be argued that our results are affected by decreased bacterial growth and/or invasion of the mutant strains as compared to the wild-type during infection, it is important to emphasize that the aim of our study was to evaluate the outcome of the host-bacteria interaction at the mucosal level and hence the effects of these mutants and their attenuated properties on the intestinal epithelial barrier in as close to physiological conditions as possible. Future studies will be directed to evaluate the molecular determinants of the remarkable effects on barrier function and cytokine production described in this manuscript.

Overall we were able to demonstrate that *S*. Typhi impairment of the epithelial barrier function occurs by modulating the sealing function of tight junctions with the consequent increase of the monolayer paracellular permeability. The mutant strains show either an attenuated or no effect on tight junction disassembly and paracellular permeability compared to the more aggressive phenotype detected in cells exposed to the wild-type strain. It is reasonable to hypothesize that the observed differences between attenuated and wild-type *S*. Typhi strains might be essential for the lack of reactogenicity and remarkable immunogenicity observed when these vaccine strains were fed to volunteers in Phase 1 and 2 clinical trials (Tacket et al., 2000b, 2004; Levine et al., 2001; Salerno-Goncalves et al., 2003, 2004; Sztein, 2007; Wahid et al., 2007, 2008, 2011, 2012; McArthur and Sztein, 2012). Another aspect of importance in intestinal mucosal defense is the capability of intestinal epithelial cells to “prime” the gut associated lymphoid tissue to possible danger caused by enteric pathogens by releasing pro-inflammatory cytokines and chemokines, including IL-8. We have demonstrated that Caco2 monolayers exposure to luminal *S*. Typhi secrete IL-8 in a polarized manner, with higher release in the basolateral side than in the luminal side at 22 h post-infection. Both HK bacteria and CM caused the release of much lower IL-8 levels, suggesting that live bacteria are more efficient in triggering danger signals to be released by enterocytes, e.g., the release of large amounts of IL-8, which in turn causes neutrophil recruitment into the lamina propria to properly face a possible enteric infection. *S*. Typhi attenuated vaccines triggered a milder IL-8 release, in keeping with their milder effects seen regarding barrier function. Noteworthy is the apical secretion of both IL-8 and IL-6.

Several studies have described the polarized secretion of these acute phase pro-inflammatory molecules (Zeillemaker et al., 1995; Carolan et al., 1997; Nasreen et al., 2001; Fahey et al., 2005; Sun et al., 2008). Their release on the “luminal” side can have significant implications. Epithelial cells represent the first site of contact bacteria have with the host and this interaction is likely to trigger a series of early events that ultimately will prevent harmful microorganisms from invading and damaging the host. The apical secretion of IL-8 and IL-6 may create a chemotactic gradient and facilitate neutrophil trans-epithelial migration to the luminal side where neutrophils can constitute a defense barrier and perform their essential function of eliminating invading microorganisms. This possibility is supported by our data showing that in the first few hours following infection IL-8 release is significantly polarized toward the apical side (Figure 9C). The basolateral release of cytokines at later times will result in the recruitment of immune cells to the lamina propria, thus playing a role in supporting and amplifying the early epithelial inflammatory response. In exploring these interesting possibilities in future studies we should take into account that expression of the Vi antigen might affect the secretion of IL-8. It has been shown that deletion of the genes associated with the regulation, biosynthesis and export of the Vi-capsular antigen (*viaB* locus) increases IL-8 expression elicited by *S*. Typhi in HEK293 cell line (Raffatellu et al., 2005). Similarly, infection of Caco2 cells with Vi^+^ *S*. Typhi produced significantly lower levels of IL-8 as compared with Vi^−^ *S*. Typhi (Sharma and Qadri, 2004). Thus, the constitutive expression of the Vi antigen might have played a role in our results with the mutant CVD 909. Future studies will address this possibility.

Overall, a better understanding of the interactions between enteric bacteria and intestinal epithelial cells is the basis for the development and improving of preventive interventions. Our results show that the intestinal mucosa is more than just a merely physical barrier against infection. Intestinal epithelial cells operate as an active extension of our innate immune system, performing a surveillance function that can specifically identify enemies and activate an offensive response to block infection.

In conclusion, the application of a multidisciplinary approach to study bacterial pathogenesis, along with the recent sequencing of entire microbial genomes have made possible discoveries that are changing the way scientists view bacterium-host interactions. Currently, research on the molecular basis of the pathogenesis of infective enteric diseases of necessity transcends established boundaries between microbiology, cell biology, intestinal pathophysiology, and immunology. Our contribution outline the need to integrate studies on bacterial pathogenesis with the host response in order to better understand the clinical outcome of bacterial enteric diseases and develop proper preventive interventions, including the development of attenuated, protective vaccines.

## Conflict of Interest Statement

Drs. M. M. Levine and M. B. Sztein are co-inventors in a patent for the development of *S. Typhoid* and *S. Paratyphoid* vaccines licensed to Bharat Biotech International Limited. The remaining authors declare no conflicting financial interests.
